# Clinical Outcomes of Surgical Revascularization in Patients Presenting with Critical Limb Ischemia and Aortic Valve Stenosis

**DOI:** 10.3390/jcdd12080292

**Published:** 2025-07-31

**Authors:** Luca Attisani, Alessandro Pucci, Matteo A. Pegorer, Luca Luzzani, Francesco Casali, Giorgio Luoni, Stefano Tanagli, Gabriele Piffaretti, Raffaello Bellosta

**Affiliations:** 1 Vascular and Endovascular Unit, Poliambulanza Foundation Hospital, 25124 Brescia, Italy; alessandro.pucci@poliambulanza.it (A.P.); matteo.pegorer@poliambulanza.it (M.A.P.); luca.luzzani@poliambulanza.it (L.L.); francesco.casali@poliambulanza.it (F.C.); giorgio.luoni@poliambulanza.it (G.L.); stefano.tanagli@poliambulanza.it (S.T.); raffaello.bellosta@poliambulanza.it (R.B.); 2 Vascular Surgery, Department of Medicine and Surgery, University of Insubria School of Medicine, 21100 Varese, Italy; gabriele.piffaretti@uninsubria.it

**Keywords:** critical limb ischemia, surgical peripheral revascularization, peripheral bypass surgery, aortic valve stenosis, lower limb ischemia

## Abstract

(1) Background: Comparison of clinical outcomes between patients with moderate-severe aortic valve stenosis and those with mild or no aortic valve stenosis undergoing surgical revascularization for critical limb threating ischemia (CLTI). (2) Methods: Single center retrospective analysis of consecutive patients undergoing surgical lower limb revascularization with femoro-distal bypass for critical ischemia between 2016 and 2022. All patients were evaluated preoperatively by echocardiographic examination and divided into two cohorts: group A with moderate-severe aortic valve stenosis (AVA-cm^2^ < or =1.5 cm^2^) and group B with mild or absent stenosis (AVA-cm^2^ > 1.5 cm^2^). Primary outcomes were major limb amputation and mortality between the two groups. The rate of major cardiovascular events (stroke, myocardial infarction, sudden cardiac death) and change in “preoperative functional status” were the secondary outcomes. Descriptive statistics for continuous variables were performed by calculating means, standard deviation (SD) medians, and interquartile range (IQR) while, for categorical variables, frequencies and percentages were performed. Intergroup comparison tests, for continuous variables, were performed by *t*-test or corresponding nonparametric tests (Mann-Whitney test) while, for categorical variables, Chi-square test was used. Evaluation of cut-offs for the variable AVA-fx-cm^2^, in terms of predictive of outcome outcomes, was calculated by ROC curves. Comparison between clinical and outcome variables was performed using logistic regression models. A total of 316 patients were analyzed and divided in two groups: 50 (16%) patients with moderate or severe aortic valve stenosis (group A) and 266 (84%) with no or mild aortic valve stenosis (AVA > 1.5 cm^2^). Patients in group A were significantly older than those in group B (78 years vs. 74 years, *p* value = 0.005); no other significant comorbidity differences were found between the two groups. The mean follow-up was 1178 days (SD 991 days; 2–3869 days). There were no statistically significant differences between group A and group B in terms of major amputation rate (20% vs. 16.5%; *p* = 0.895) and overall mortality (48.0% vs. 40.6%; *p* = 0.640). In the total cohort, the statistically significant variables associated with the major amputation were systemic perioperative complication (OR 5.83, 95% CI: 2.36, 14.57, *p* < 0.001), bypass-related complication within 30 days of surgery (OR 2.74, 95% CI: 1.17, 6.45, *p* = 0.020), surgical revascularization below the knee (OR 7.72, 95% CI: 1.53, 140.68, *p* = 0.049), and the presence of a previous cardiovascular event (OR 2.65, 95% CI: 1.14, 6.26, *p* = 0.024). In patients undergoing surgical revascularization for CLTI, no significant difference in major amputation rate and overall mortality was found between subjects with mild or no aortic valve stenosis and those with moderate/severe stenosis. As expected, overall mortality was higher in older patients with worse functional status. A significantly higher rate of limb amputation was found in those subjects undergoing subgenicular revascularization, early bypass failure, or previous cardiovascular event.

## 1. Introduction

There are more than 200 million people affected by critical threatening limb ischemia (CTLI) worldwide, with a growth rate of 23.5% between 2000 and 2010 due to aging of the general population and the epidemiological increase in risk factors, particularly diabetes mellitus [1]. The increase in the incidence of CTLI parallels the increase in the incidence of aortic valvular stenosis; the prevalence of this condition is estimated to be 2% in the population aged 65 to 75 years, 3% in the population aged 75 to 85 years, and around 4% in the population over 85 years of age [2,3]. Often the two conditions coexist, with a reported prevalence of CTLI in patients submitted to Transcatheter Aortic Valve Implantation (TAVI) up to 34% [4] associated with a high of in-hospital mortality [5]. However, this aspect is not underlined by the recent European Guidelines of management of CTLI [6]. The purpose of this work was to compare the clinical outcomes between patients with moderate-severe aortic valve stenosis and those with mild or no aortic valve stenosis and with critical lower extremity ischemia undergoing surgical revascularization.

## 2. Materials and Methods

### 2.1. Study Design

This is a single-center, retrospective, observational cohort study of prospectively collected patients. All patients who underwent infrainguinal surgical revascularization were identified: post-hoc analysis of the institutional database identified those who underwent surgical lower limb revascularization with femoro–supra- or sub-articular bypass for CTLI from November 2016 to April 2022. All patients provided written informed consent, and each center’s institutional review board approved the present study in accordance with the national policy on the retrospective analysis of anonymized data and the Italian privacy act.

Inclusion criteria were:

- CTLI with Rutherford stage 3, 4, 5, or 6 [6].

- Patients submitted to peripheral bypass starting from the infrainguinal region (common femoral, superficial femoral, or popliteal artery) and landing supra- or sub-articular (popliteal artery or tibial vessels).

Exclusion criteria were:

-Patients treated with endovascular revascularization and those submitted to TAVI before surgical revascularization or during follow-up.

### 2.2. Cardiac Evaluation

Each patient underwent preoperative cardiologic examination at the cardiac lab at our institution. Transthoracic echocardiogram was performed by cardiologists with more than 10 years of experience.

### 2.3. Surgical Details and Follow-Up Protocol

Patients were treated according to the European guidelines, taking into account the Global Limb Anatomic Staging System (GLASS classification) [6]. The great saphenous vein, when available, was the material of choice for revascularization; in case of its unavailability, prosthetic material (PTFE or Dacron) with interposition of vein Linton patch at the distal anastomosis was performed. At the end of the intervention, completion angiography or duplex ultrasound was routinely achieved. Postoperative antithrombotic treatment consisted of single or double antiplatelet treatment or oral anticoagulation; it was determined on the basis of the surgeon’s preference as well as according to the patient’s comorbidities and risk factors, or it was driven by some particular technical aspects during the intervention. Clinical and instrumental follow-up with echo-color Doppler was performed at one, three, six, and 12 months, and annually thereafter. At each follow-up visit, functional status and degree of disability was assessed and compared with the preoperative situation. Patients with trophic lesions, gangrene, or amputation were followed at a dedicated outpatient vulnology clinic, performed by professional nurses, and supervised by medical staff, until the subjects’ complete recovery, death, or major amputation surgery.

### 2.4. Definitions

The extent of aortic valve stenosis was assessed according to the criteria of the European Society of Cardiology [7] Table 1. Patients were divided into two groups: group A, those presenting with moderate-severe aortic valve stenosis (AVA-cm^2^ < or =1.5 cm^2^), and group B, presenting mild or no stenosis (AVA-cm^2^ > 1.5 cm^2^). All subjects were classified on the basis of functional status and degree of disability according to the classification proposed by the American Society of Vascular Surgery (SVS) [8] and outlined in Table 2.

The total days of each patient’s hospital stay related to the bypass procedure was taken into account. Perioperative complications, considered temporally to have occurred within the first 30 days after surgery, were divided according to the type of event into systemic (cardiac, respiratory, renal, neurological, thrombo-embolic, allergic) or bypass-related (including peri-anastomotic stenosis, thrombosis, pseudoaneurysms, arteriovenous fistulae, infection, and need for major amputation due to acute bypass occlusion and non-revascularization of the limb). In the follow-ups after 30 days, only late bypass-related complications (including peri-anastomotic stenosis evidenced on postoperative echo-color Doppler follow-up, thrombosis of the same, pseudoaneurysms, arteriovenous fistulas, or infections) were considered.

Rates of major limb amputation and incidence of major cardiovascular events, defined as occurrence of cerebral stroke, myocardial infarction, and sudden cardiac death, were calculated, along with number of days elapsed from the date of surgery.

Mortality was investigated by consulting demographic information afferent to the Lombardy Region SISS service or by telephone consultation with family members. Among the patients who died, it was specified whether death was from cardiovascular or non-cardiovascular causes. In case of doubt or inability to find this information, the cause of death was listed as “other non-cardiovascular cause”.

### 2.5. Outcomes

Primary outcomes were major amputation rate and mortality rate between the two groups. Secondary outcomes included the rate of major cardiovascular events, late bypass-related complications (including peri-anastomotic stenosis evidenced on postoperative echo-color Doppler follow-up, thrombosis of the same, pseudoaneurysms, arteriovenous fistulas, or infections) and change in “preoperative functional status”.

### 2.6. Statistical Analysis

Clinical data were recorded prospectively and tabulated in a Microsoft Excel (Microsoft Corp., Redmond, WA, USA) database; statistical analysis was performed by means of SPSS 24.0 for Windows (IBM SPSS Inc., Chicago, IL, USA). Categorical variables were presented as frequencies and percentages, and continuous variables were presented as mean ± standard deviation or median and ranges, depending on data distribution. Continuous variables were analyzed with the X^2^ and Fisher’s exact test, when necessary. Independent samples Student’s *t*-test was used for continuous variables, and the Wilcoxon signed-rank test was used to evaluate the difference in ankle-brachial index measurement before and after intervention. Associations that yielded a *p* value of less than 0.20 in the univariate screen were then included in a forward Cox regression analysis. The strength of the association of variables with PVGI was estimated by calculating the hazard ratio and 95% confidence intervals (Cis; significance criteria of 0.25 for entry and 0.05 for removal). Evaluation of cut-offs for the variable AVA-fx-cm^2^, in terms of predictive of outcome outcomes, was calculated by ROC curves. All reported *p* values were two-sided; a *p* value of less than 0.05 was considered significant.

## 3. Results

### 3.1. Clinical Profile, Anatomic Characteristics, and Surgical Details

A total of 158 patients were analyzed, of whom the majority were male (111, 70.2%), 25 (15.8%) of them belonging to group A, and 133 (84.2%) to group B. Patients in group A were found to be significantly older than those in group B (median age 78 [65.81] vs. 74 [76.83], *p* value = 0.005). Demographic characteristics and comorbidities of the population can be consulted in Table 3. Clinical profile and surgical details are listed on Table 4.

### 3.2. Bypass Related Outcomes

Median time in days of hospitalization was 7 for group A (IQR 5.75, 9.00) and 6 for group B (IQR 5.00, 9.00, *p* = 0.467). Mean follow-up was 1178 days, (SD 991 days). In total, we observed 51 (32.2%) bypass-related complications within 30 days from intervention. A total of 27 (17%) major amputations were performed at follow-up. Graft related complications are listed in Table 5. Among patients who had at least four complications, there were significantly more in group A than in group B (*p* = 0.010). In general, in group A, more patients had more than two complications compared to patients in group B.

The presence of moderate-severe aortic valve stenosis does not appear to be associated with major amputation (OR 0.76, *p* = 0.638). The only variable with statistical significance was the time elapsed (in days) between the date of revascularization surgery and the date of major amputation: 449 days in group A vs. 100 in group B (*p* = 0.016). Other variables associated with increased risk of amputation are highlighted in Table 6.

### 3.3. Clinical Outcomes

Clinical outcomes in terms of cardiovascular and other cause mortality, postoperative functional recovery, and incidence of postoperative major cardiovascular events are listed in Table 7.

**Figure 1:**
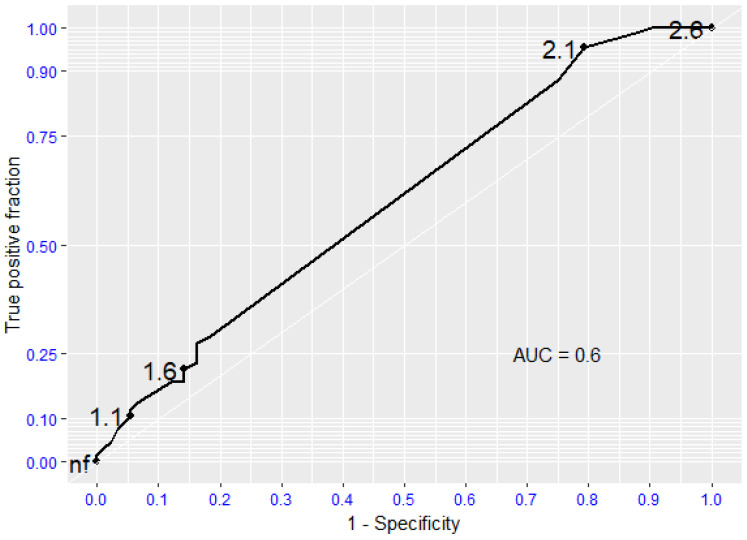
Receiving Operating Characteristic (ROC curve) regarding global mortality. AUC (Area Under the Curve).

ROC analysis documented that the presence of moderate-severe aortic valvular stenosis is not a reliable predictor of death or survival in patients undergoing surgical lower extremity revascularization (AUC 0.6; 95% CI: 0.52, 0.68). The absence of aortic valve stenosis is a statistically significant predictor of survival (AUC 0.8–0.95) (Figure 1).

Significant predictors of overall mortality were age (OR = 1.07, 95% CI: 1.03, 1.11, *p* < 0.001), systemic complication within 30 days (OR 3.83, 95% CI: 1.69, 9.16, *p* = 0.002), and pre-operative functional status “need some assistance” (OR 8.57, 95% CI: 3.30, 24.80, *p* < 0.001) and “required total assistance” (OR 20.57, 95% CI: 5.10, 109.9, *p* < 0.001).

**Figure 2:**
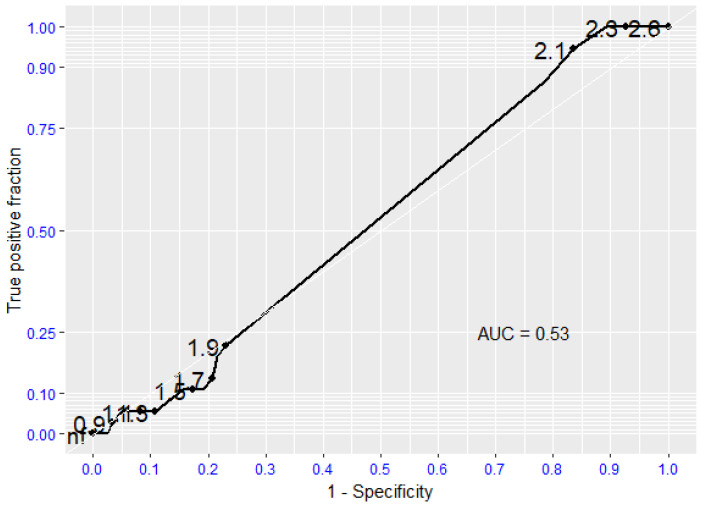
Receiving Operating Characteristic (ROC curve) regarding Major Adverse Events (MAE). AUC (Area Under the Curve).

Regarding survival following Major Adverse Event (myocardial infarction, ICTUS, sudden cardiac death), there is no evidence that moderate-severe aortic valve stenosis is a predictive risk factor (*p* = 0.934), while the absence or presence of mild aortic valvular stenosis was predictive of the absence of such events, with sensitivity and specificity values for survival following major cardiovascular complications of 0.95 and 0.8, respectively (Figure 2).

Significant predictors of cardiovascular mortality were age (OR 1.05, 95% CI: 1.01, 1.09, *p* = 0.028), systemic complication within 30 days (OR 3.72, 95% CI: 1.60, 8.67, *p* = 0.002), and pre-operative functional status “need some assistance” (OR 10.37, 95% CI: 3.18, 47.03, *p* < 0.001).

The presence of moderate-severe aortic valve stenosis does not appear to be associated with changes in preoperative functional status (OR 0.45, *p* = 0.111). Variables associated with changes in preoperative functional status can be consulted in Table 8.

## 4. Discussion

Despite a higher rate of mortality and complications in patients undergoing TAVI and suffering from CTLI than in nonarterial patients being well documented [9,10], there is lack of evidence in the literature of clinical outcomes of patients submitted to surgical revascularization for CTLI and suffering from severe aortic valve stenosis.

One explanation could be the increasing application of endovascular revascularization techniques burdened by a low perioperative risk that does not require cardiologic preoperatory investigations [11]. In addition, echocardiogram is not always performed, due to the rapidly evolving nature of disease in these categories, and thus of patients that needing a treatment as quickly as possible. Secondly, with the advent of TAVI, which has made it possible to extend the therapeutic indication even to patients with severe comorbidities considered inoperable with traditional technique, the encounter of patients with untreated severe aortic valve stenosis is increasingly rare [12].

As expected, the only significant difference in demographic data between the two groups was related to age; patients with moderate-severe aortic valvular stenosis were significantly older than subjects afferent to the comparison group. These findings are in line with other data reported in the literature [2,3]. Age and severe aortic valve stenosis were also associated, albeit non-significantly, with a greater rate of ischemic foot impairment (Rutherford grade 4) than the control group.

The main finding of this study was that moderate-severe aortic valve stenosis was not significantly associated with a higher rate of amputation or worse survival than patients without or mild valve disease. Previous works have not found a difference in terms of limb survival after surgical revascularization between octogenarians and non-octogenarian patients [13,14]. Considering those patients with moderate-severe aortic valve stenosis are older than the control group, we can assume that our findings are in line with others. However, even if not significant, patients in group A tended to have a higher rate of bypass complications than the control group. Many factors impact the outcome of lower extremity bypass. It has long been established that veins are the ideal conduit for lower extremity revascularization. With aging, venous compliance decreases, especially in the lower extremities, which can make vein graft in the elderly more susceptible to early stenosis and graft failure [15].

However, interestingly, we found that the time elapsed in days between the date of revascularization and the date of major amputation was significant longer in the group with moderate-severe stenosis than the group with mild or no stenosis (499 days vs. 100 days, *p* = 0.016). This apparently counter-intuitive finding could be explained as a bias arising from the comparison of two numerically heterogeneous groups of patients.

Regarding mortality data, among patients who died of cardiovascular causes, those with moderate-severe aortic valve stenosis had a shorter postoperative survival time in days than patients with mild or without significant valve stenosis. This finding confirms what is already evident in the literature, that patients with high-grade aortic valve disease have higher mortality rates than those without valve stenosis [16].

In the group of patients with moderate-severe stenosis, the percentage that died from non-cardiovascular causes was higher (32.0% vs. 15.8%). Among the causes, in addition to advanced age and major comorbidities, the SARS-CoV-2 pandemic in the early 2020s caused many to die from COVID19-related respiratory causes, which may have played a role in this study [17].

A very interesting aspect worthy of further study is the change in functional status after revascularization surgery. Although not significant, full recovery of autonomy in the performance of daily activities was observed at a higher percentage within the cohort of patients with mild or absent stenosis, 43.9%, compared with 29.2% in the cohort of moderate-severe aortic valve stenosis. Our work confirmed data from that of Taylor et al., who showed that among the most important predictors of failure to improve functional outcome appeared to be “impaired ambulatory ability” at presentation, along with the presence of dementia [18].

## 5. Limitations

This study has several limitations, first being the retrospective and single design of the work; therefore, the possibility of biased analysis cannot be excluded.

Secondarily, due to limited data, it was not possible to calculate ROC curve. We performed univariate logistic model to verify which variables were statistically associated with major amputation and variation of functional status. Our study’s limited event rate has dual implications: it may suggest effective clinical practice, but also, the eventual absence of statistically significant differences could reflect a type II error.

Lastly, many of our patients were lost to follow-up. Notwithstanding these limitations which may not allow for generalizability of our findings, our data compares well with the available literature owing to the very recent nature of the cohort and homogeneity protocol and follow-up within our center, as well as data validation by official health documents.

## 6. Conclusions

The results obtained from this research showed that patients submitted to surgical peripheral revascularization for CLTI and concomitantly affected by moderate-severe aortic valve stenosis have similar outcomes in term of amputation and mortality rate than patients with mild or without aortic valve stenosis.

However, this cohort of patients was overall older, frail, and had temporally more “aggressive” cardiovascular mortality than the control group.

This study showed that surgical treatment of CTLI, in addition to bringing a significant reduction or complete disappearance of pain at rest and ensuring healing of trophic lesions and/or minor amputation site, is associated with improved postoperative functional status in patients without aortic valve disease. Further large-scale prospective studies are needed to confirm these data.

## Figures and Tables

**Table 1:** Classification of aortic valve stenosis (AS).

Echo Parameters	Sclerosis	Mild AS	Moderate AS	Severe AS
Peak velocity, m/s	<2.5	2.5–3	3–4	>4
Mean gradient, mmhg	Normal	<20	20–40	40
AVA, cm^2^	Normal	≥1.5	1–1.5	<1

**Table 2:** Pre-operatory functional status and disabilities. ADL: Activities of Daily Living.

Pre-Operatory Functional Status	
0	No impairment
1	Impaired, but able to carry out ADL without assistance
2	Needs some assistance to carry out ADL or ambulatory assistance
3	Requiring total assistance for ADL or nonambulatory

**Table 3:** Demographic data and comorbidities. BMI: Body Mass Index, COPD: Chronic Obstructive Pulmonary Disease, MI: Myocardial Infarction, EF: Cardiac Ejection Fraction, ADL: Activities of Daily Living.

Variable	Level	Group B	Group A	*p*-Value
		*n* = 133	*n* = 25	
Sex (%)	Female	39 (29.3)	8 (32.0)	0.976
	Male	94 (70.7)	17 (68.0)	
Age (median, [IQR])		74.00 [65.00, 81.00]	78.00 [76.00, 83.00]	0.005
Vmax (median [IQR])		1.36 [1.17, 1.65]	2.89 [2.60, 3.19]	<0.001
Mean Gp (median [IQR])		3.80 [2.90, 5.60]	18.42 [13.16, 24.04]	<0.001
Weight, kg (median [IQR])		70.00 [58.00, 80.00]	70.00 [55.00, 78.00]	0.911
Height, m (median [IQR])		1.69 [1.62, 1.75]	1.67 [1.64, 1.70]	0.561
BMI (median [IQR])		24.69 [22.22, 26.67]	24.80 [22.40, 27.66]	0.971
Smoking habits (%)	No, past	92 (69.2)	19 (76.0)	0.655
	Yes	41 (30.8)	6 (24.0)	
Diabetes (%)	No	73 (54.9)	11 (44.0)	0.434
	Yes	60 (45.1)	14 (56.0)	
Hypertension (%)	No	19 (14.3)	2 (8.0)	0.597
	Yes	114 (85.7)	23 (92.0)	
Renal function impairment (%)	No impairment or mild	116 (87.2)	24 (96.0)	0.355
	Severe or pre-terminal	17 (12.8)	1 (4.0)	
Dislipidemia (%)	No	34 (25.6)	6 (24.0)	1.000
	Yes	99 (74.4)	19 (76.0)	
Cardiac status (%)	Asymptomatic or previous MI (>6 months) or silent	86 (64.7)	15 (60.0)	0.827
	Recent MI (<6 mesi), arytmia, angina, reduction of EF	47 (35.3)	10 (40.0)	
COPD (%)	No	61 (45.9)	11 (44.0)	1.000
	Yes	72 (54.1)	14 (56.0)	
Stroke (%)	No	115 (86.5)	23 (92.0)	0.663
	Yes	18 (13.5)	2 (8.0)	
Pre-operatory functional status (%)	No impairment	39 (29.3)	4 (16.0)	0.479
	Impaired, but able to carry out ADL without assistance	44 (33.1)	8 (32.0)	
	Needs some assistance to carry out ADL or ambulatory assistance	38 (28.6)	10 (40.0)	
	Requiring total assistance for ADL or nonambulatory	12 (9.0)	3 (12.0)	

**Table 4:** Clinical presentation, type of intervention and graft material.

Variable	Level	Group B	Group A	*p*-Value
Side *n* (%)	Right	67 (50.4)	8 (32.0)	0.142
	Left	66 (49.6)	17 (68.0)	
Rutherford Scale (%)	3—Severe claudication	10 (7.5)	2 (8.0)	0.157
	4—Ischemic rest pain	34 (25.6)	4 (16.0)	
	5—Minor tissue lost	83 (62.4)	15 (60.0)	
	6—Major tissue lost	6 (4.5)	4 (16.0)	
Level of revascularization *n* (%)	Above the knee	26 (19.5)	5 (20.0)	1.000
	Below the knee	107 (80.5)	20 (80.0)	
Graft material, prosthesis *n* (%)	No	47 (35.3)	9 (36.0)	1.000
	Yes	86 (64.7)	16 (64.0)	
Graft material, great saphenous vein *n* (%)	No	64 (48.1)	10 (40.0)	0.597
	Yes	69 (51.9)	15 (60.0)	

**Table 5:** Graft related complications and amputation rate.

Variable	Level	Group B	Group A	*p*-Value
Bypass-related complications within 30 days *n* (%)	No	91 (68.4)	16 (64.0)	0.841
	Yes	42 (31.6)	9 (36.0)	
Patency, *n* (%)	No	41 (31.5)	9 (36.0)	0.839
	Yes	89 (68.5)	16 (64.0)	
Restenosis, *n* (%)	No	87 (97.8)	16 (100.0)	1.000
	Yes	2 (2.2)	0 (0.0)	
First complication after 30 days *n* (%)	No	77 (57.9)	12 (48.0)	0.487
	Yes	56 (42.1)	13 (52.0)	
Second complication after 30 days *n* (%)	No	112 (84.2)	21 (84.0)	1.000
	Yes	21 (15.8)	4 (16.0)	
Third complication after 30 days *n* (%)	No	124 (93.2)	21 (84.0)	0.252
	Yes	9 (6.8)	4 (16.0)	
Fourth complication after 30 days *n* (%)	No	132 (99.2)	22 (88.0)	0.010
	Yes	1 (0.8)	3 (12.0)	
Mean number of complications after 30 days [SD]		0.65 [0.89]	0.96 (1.34)	0.429
Major amputation (%)	No	111 (83.5)	20 (80.0)	0.895
	Yes	22 (16.5)	5 (20.0)	
Time between intervention and major amputation in days (median, [IQR])		100.00 [64.50, 283.25]	449.00 [432.00, 1144.00]	0.016

**Table 6:** Univariate logistic model for “Major Amputation”, AVA (Aortic Valve Area), MI (Myocardial Infarction).

	Univariate Logistic Model “Major Amputation”		
Predictors	Odds Ratio	OR 95% CI	*p*-Value
AVA	0.76	(0.27, 2.49)	0.638
Age	1.02	(0.97, 1.07)	0.420
Complications within 30 days [yes vs. no]	5.83	(2.36, 14.57)	<0.001
Functional status [Impaired, but able vs. not impaired]	2.42	(0.87, 25.67)	0.109
[Needs some assistance vs. not impaired]	10.37	(1.72, 45.82)	0.016
[Requiring total assistance vs. not impaired]	6.67	(1.91, 79.39)	0.010
Graft related complications [yes vs. no]	2.74	(1.17, 6.45)	0.020
Bypass length	7.72	(1.53, 140.68)	0.049
Cardiac status [recent MI vs. asymptomatic]	2.65	(1.14, 6.26)	0.024

**Table 7:** Clinical outcomes between the two groups. MAE: Major Adverse Event, MI: Myocardial Infarction.

Variable	Level	Group B	Group A	*p*-Value
Major Adverse Event (MAE) (%)	No	79 (59.4)	13 (52.0)	0.142
	Cardiovascular event (MI/stroke/Sudden Cardiac death)	33 (24.8)	4 (16.0)	
	Death by other causes or unknown	21 (15.8)	8 (32.0)	
Death, *n* (%)	No	79 (59.4)	13 (52.0)	0.640
	Yes	33 (24.8)	12 (48.0)	
Cardiovascular death, *n* (%)	No	100 (75.2)	21 (84.0)	0.486
	Yes	33 (24.8)	4 (16.0)	
Times between intervention and any MAE in days (median [IQR])		936 [238, 1478]	792.50 [159.25, 1431.50]	0.673
Time between intervention and death in days (mean; median [IQR])		961; 744.50 [244.50, 1475.25]	893; 559 [165.25, 1431.50]	0.630
Time between intervention and cardiovascular death in days (mean; median [IQR])		822; 341 [150, 1467]	358; 181 [86.75, 452.25]	0.221
Postoperative functional status, *n* (%)	No impairment	58 (43.9)	7 (29.2)	0.157
	Impaired, but able to carry out ADL without assistance	20 (15.2)	7 (29.2)	
	Needs some assistance to carry out ADL or ambulatory assistance	23 (17.4)	2 (8.3)	
	Requiring total assistance for ADL or nonambulatory	31 (23.5)	8 (33.3)	

**Table 8:** Univariate logistic model for variation of functional status, AVA (Aortic Valve Area), MI (Myocardial Infarction).

	Univariate Logistic Model for Variation of Functional Status		
Predictors	Odds Ratio	OR 95% CI	*p*-Value
AVA	0.45	(0.16, 1.16)	0.111
Age	1.08	(1.04, 1.13)	<0.001
Complications within 30 days [yes vs. no]	3.52	(1.43, 10.04)	0.010
Functional status [Impaired, but able vs. not impaired]	3.02	(1.28, 7.49)	0.013
[Needs some assistance vs. not impaired]	10.62	(6.09, 51.17)	<0.001
[Requiring total assistance vs. not impaired]	40.72	(6.98, 782.41)	<0.001
Graft related complications [yes vs. no]	1.82	(0.91, 3.77)	0.095
Bypass length	2.52	(1.13, 5.82)	0.026
Cardiac status [recent MI vs. asymptomatic]	2.12	(1.07, 4.30)	0.033
Rutherford scale	2.24	(1.38, 3.77)	0.014

## Data Availability

The data underlying this article will be shared on reasonable request to the corresponding author.
